# Potential, Pitfalls, and Future Directions for Remote Monitoring of Chronic Respiratory Diseases: Multicenter Mixed Methods Study in Routine Cystic Fibrosis Care

**DOI:** 10.2196/54942

**Published:** 2024-08-06

**Authors:** Martinus C Oppelaar, Yvette Emond, Michiel A G E Bannier, Monique H E Reijers, Hester van der Vaart, Renske van der Meer, Josje Altenburg, Lennart Conemans, Bart L Rottier, Marianne Nuijsink, Lara S van den Wijngaart, Peter J F M Merkus, Maud Heinen, Jolt Roukema

**Affiliations:** 1 Department of Pediatric Pulmonology Amalia Children's Hospital Radboud University Medical Center Nijmegen Netherlands; 2 IQ Health Science Department Radboud University Medical Center Nijmegen Netherlands; 3 Department of Paediatric Pulmonology MosaKids Children’s Hospital Maastricht University Medical Centre+ Maastricht Netherlands; 4 Department of Pulmonology Radboud University Medical Center Nijmegen Netherlands; 5 Department of Pulmonary Diseases University Medical Center Groningen University of Groningen Groningen Netherlands; 6 Department of Pulmonology Haga Teaching Hospital The Hague Netherlands; 7 Department of Respiratory Medicine Amsterdam University Medical Centers University of Amsterdam Amsterdam Netherlands; 8 Department of Respiratory Medicine Maastricht University Medical Centre+ Maastricht Netherlands; 9 Division of Respiratory & Age-related Health Department of Respiratory Medicine NUTRIM Institute of Nutrition and Translational Research in Metabolism Maastricht Netherlands; 10 Department of Pediatric Pulmonology and Pediatric Allergology University Medical Center Groningen Beatrix Children's Hospital, University of Groningen Groningen Netherlands; 11 Groningen Research Institute for Asthma and COPD University Medical Center Groningen University of Groningen Groningen Netherlands; 12 Haga Teaching Hospital Juliana Children's Hospital The Hague Netherlands

**Keywords:** telemonitoring, digital health, chronic respiratory diseases, telespirometry, interviews, mixed methods, qualitative study, remote monitoring, evaluation, cystic fibrosis, pediatrics, mixed method, observational study, health care professionals, semistructured interview, psychosocial, clinicians, researchers, policy makers, telehealth

## Abstract

**Background:**

The current literature inadequately addresses the extent to which remote monitoring should be integrated into care models for chronic respiratory diseases (CRDs).

**Objective:**

This study examined a remote monitoring program (RMP) in cystic fibrosis (CF) by exploring experiences, future perspectives, and use behavior over 3 years, with the aim of developing future directions for remote monitoring in CRDs.

**Methods:**

This was a mixed methods, multicenter, observational study in 5 Dutch CF centers following a sequential explanatory design. Self-designed questionnaires using the technology acceptance model were sent out to people with CF who had a minimum of 12 months of experience with the RMP and local health care professionals (HCPs). Questionnaire outcomes were used to inform semistructured interviews with HCPs and people with CF. Qualitative findings were reported following the COREQ (Consolidated Criteria for Reporting Qualitative Research) checklist. Anonymous data on use frequency of all people with CF were analyzed.

**Results:**

Between the second quarter of 2020 and the end of 2022, a total of 608 people with CF were enrolled in the program, and a total of 9418 lung function tests and 2631 symptom surveys were conducted. In total, 65% (24/37) of HCPs and 89% (72/81) of people with CF responded to the questionnaire, and 7 HCPs and 12 people with CF participated in semistructured interviews. Both people with CF and HCPs were positive about remote monitoring in CF care and found the RMP a good addition to daily care (people with CF: 44/72, 61%; HCPs: 21/24, 88%). Benefits ranged from supporting individual patients to reducing health care consumption. The most valued monitoring tool was home spirometry by both people with CF (66/72, 92%) and HCPs (22/24, 92%). Downsides included the potential to lose sight of patients and negative psychosocial effects, as 17% (12/72) of people with CF experienced some form of stress due to the RMP. A large majority of people with CF (59/72, 82%) and HCPs (22/24, 92%) wanted to keep using the RMP in future, with 79% (19/24) of HCPs and 75% (54/72) of people with CF looking forward to more replacement of in-person care with digital care during periods of well-being. Future perspectives for the RMP were centered on creating hybrid care models, personalizing remote care, and balancing individual benefits with monitoring burden.

**Conclusions:**

Remote monitoring has considerable potential in supporting people with CF and HCPs within the CF care model. We identified 4 practice-based future directions for remote monitoring in CF and CRD care. The strategies, ranging from patient driven to prediction driven, can help clinicians, researchers, and policy makers navigate the rapidly changing digital health field, integrate remote monitoring into local care models, and align remote care with patient and clinician needs.

## Introduction

### Background

Chronic respiratory disease (CRD) management needs to undergo rapid changes to respond to growing challenges in the coming decades. CRDs are one of the largest contributors to the global noncommunicable disease burden, and their prevalence is increasing worldwide [1]. At the same time, health systems are under pressure due to rising health care costs and health worker shortages. Consequently, there are increasing calls for innovative solutions such as remote monitoring to relieve some of this burden and ensure health care continuity for patients with asthma, chronic obstructive pulmonary disease, and other CRDs [2]. The COVID-19 pandemic has shown that remote monitoring in CRD management can be feasible and effective, but there are still uncertainties regarding to what extent remote monitoring should be integrated into existing care models [3-5].

The field of cystic fibrosis (CF) provides a perfect case study to analyze this problem. CF is an autosomal recessive hereditary disease caused by a defect in the gene coding for the CF transmembrane conductance regulator (CFTR) protein [6]. CF is a multisystem disease, but progressive pulmonary deterioration due to chronic inflammation and recurrent pulmonary infections is the most frequent cause of morbidity and mortality for people with CF [6]. Up until recently, the median age of mortality was between 30 and 40 years but is now rapidly increasing due to new CFTR protein–modulating drugs [6]. These drugs specifically target the cellular defect underlying CF and lead to significant clinical improvements that will result in a larger adult CF population in the near future [7,8]. Moreover, CF will more closely resemble other CRDs as pulmonary deterioration is slowed down significantly [9]. Care models need to quickly adjust to these changes, potentially with digital solutions [9]. Therefore, the changes we witness in digital health in CF today could provide valuable guidance for the implementation of digital health in the wider field of CRD management.

Until now, a prominent focus of the literature on remote monitoring in CF has been the early identification of pulmonary exacerbations using remote monitoring. Some studies have shown that this may allow for earlier identification of pulmonary exacerbations at a population level, but there is no evidence that remote monitoring also improves clinical outcomes or quality of life for individual patients [10]. Moreover, although remote symptom and spirometry monitoring appears highly feasible, adequate uptake of remote lung function monitoring is rarely sustained over time [10,11]. As a result, the current literature now calls for a different direction, with increasing attention to evidence for improvements in other domains such as health system strengthening, patient empowerment, and workload of health care professionals (HCPs). At the same time, there is a pressing need for more guidance on the integration of remote monitoring into existing CF care models [5,8].

### Objectives

This study is part of the Airlift-CF project, which provides remote care for patients with asthma and CF in the Netherlands [12-14]. Over 40% of all Dutch people with CF in 5 Dutch CF centers are using this program in their regular care to monitor symptoms and lung function at home. The aims of this study were to determine the role of remote monitoring in CF care models by exploring experiences, future perspectives, and actual remote monitoring program (RMP) use during the 3 years after the onset of the COVID-19 pandemic and develop future directions for remote monitoring in CF and other CRDs. The findings of this study will help researchers align their efforts with clinical needs and will empower patients, HCPs, and policy makers to make informed decisions about the use of remote monitoring in routine care within different contexts.

## Methods

### Study Design

This was a mixed methods, multicenter, observational study that followed a sequential explanatory design (ie, quantitative analyses were followed by qualitative analyses to provide in-depth explanations of the findings) guided by the Mixed Methods Appraisal Tool 2018 [15]. The Strengthening the Reporting of Observational Studies in Epidemiology checklist was used to design and report this study (Multimedia Appendix 1). The study was conducted in 5 of the 7 Dutch CF centers (Radboud University Medical Center; University Medical Center Groningen; Maastricht University Medical Center+; Academic Medical Center, Amsterdam; and Haga Hospital, The Hague).

### eHealth Program

The RMP for CF was introduced in March 2020 to provide health care continuity for people with CF in response to the COVID-19 pandemic and was based on our preexisting RMP for pediatric asthma. No formal implementation project could be developed during this turbulent period. Instead, implementation was guided by our experience in implementation of remote monitoring for pediatric asthma [16]. The RMP is used to (1) monitor disease symptoms using a 7-item modified Fuchs questionnaire [17], (2) monitor lung function using a Bluetooth-connected portable spirometer (Spirobank Smart; Medical International Research), and (3) facilitate easy and secure patient-HCP contact. Further details are described in Multimedia Appendix 2 [12-14].

### Participants

Both people with CF and HCPs were asked to participate in this study. People with CF were eligible for inclusion if they were aged ≥6 years and had used the RMP for at least 12 months. The criterion for experience was chosen because little to no evidence exists on the use of remote monitoring for CF for >12 months. Therefore, this study aimed to focus explicitly on the experiences and perspectives of long-term users as our previous experiences show that adaption and habituation to remote monitoring occurs over longer periods*.* Parents of younger children who were unable to participate in this study themselves were asked to provide their experiences instead. People with CF or their parents were invited to participate by their HCPs through the RMP when they met the inclusion criteria. HCPs (medical doctors and nurses) working with the RMP were also invited to participate. No sample size calculation was performed as this was not possible for this exploratory study. Recruitment of participants started in March 2022 and was scheduled to last 6 months.

### Questionnaires

Participants received self-designed questionnaires based on the themes of perceived usefulness, perceived ease of use, intention to use, and use behavior from the technology acceptance model (TAM) [18,19]. Most questions were closed ended (ie, 5-item Likert scale). Some questions were open ended and focused on experienced advantages or disadvantages, suggestions for future use of the RMP, and incentives or disincentives (Multimedia Appendix 3). The content, relevance, and language of the questions were scrutinized by patient representatives of the Dutch CF Foundation and a small group of people with CF and HCPs. We used a modified 10-item System Usability Scale tailored to the study population to quantify perceived ease of use (Multimedia Appendix 3) [20,21]. Questionnaire invitations were sent via email, with automatic reminders after 1, 2, and 4 weeks.

### Interviews

Semistructured interviews were performed to further explore and substantiate questionnaire results. The coauthors (MCO, YE, MH, PJFMM, and JR) discussed questionnaire results to reach a consensus on which questionnaire results warranted further investigation during the qualitative part of this study. These factors included (1) the relevance of the findings for clinical practice, (2) the unexplained ambiguity of the results (eg, differences between HCPs and people with CF or varying opinions within subgroups), (3) the potential impact of the findings on people with CF and HCPs, and (4) the frequency of themes that emerged in open-ended questions. After the identification of subjects was completed, interview guides were designed using the TAM framework and complementary domains from the work by Flottorp et al [18,19,22] (the full translated interview guides are available in Multimedia Appendix 4).

For each age group (6-12 years, 12-16 years, and >16 years), people with CF who had indicated interest in interviews in the questionnaire were invited randomly via email. Selection by age groups was chosen because age is an important factor in the focus and organization of CF care—care for younger children tends to be more focused on development, whereas care for adult people with CF is more focused on pulmonary health. Therefore, perspectives and experiences will likely differ between these groups. Moreover, by including different age groups, we also allowed for the inclusion of parents of people with CF, who likely have their own unique experiences and perspectives, hence providing a more valid representation of the target population. The coauthors recruited HCPs for interviews from their teams through purposive sampling based on experience and availability. Recruitment continued until data saturation was reached within subgroups. Interviews were held by video in Dutch for up to 60 minutes; moderated by the first author (MCO), who has a medical background; and audio recorded. Verbatim transcription of the recordings was carried out by a professional service.

### Data on RMP Use

Anonymous data on frequency of use of all RMP users were extracted from January 2020 to December 2022. We described the monthly enrollment rate of participants over time, the monthly lung function rates, and the monthly symptom survey rates.

### Data Analysis

Quantitative analyses were performed using SPSS Statistics (version 27; IBM Corp), and qualitative analysis of the interviews was performed using ATLAS.ti (version 22.0.11; ATLAS.ti Scientific Software Development GmbH). Questionnaire results were summarized for HCPs and people with CF separately. Results of equivalent questionnaire questions were compared between these subgroups using the Fisher exact test. Open-ended questionnaire results were analyzed using open coding (MCO) and categorized into subthemes that emerged (MCO, PJFMM, and JR).

Transcripts were analyzed independently using open coding by MCO and YE. Themes that emerged were categorized into predefined themes from the TAM or into new themes. The codes and analyses were discussed until a consensus was reached (MCO, YE, and MH). Findings were reported following the COREQ (Consolidated Criteria for Reporting Qualitative Research; Multimedia Appendix 5 [23]) checklist.

### Ethical Considerations

Local ethical committees waived formal approval considering the negligible burden of participation and absence of imposed risks (file number for local ethical committee Arnhem-Nijmegen region: 2021-13214). All eligible people with CF and their parents provided informed e-consent on the RMP before participation in the study in accordance with the Dutch Central Committee on Research Involving Human Subjects guidelines. All data were pseudonymized using an encrypted code that was only accessible to HCPs directly involved in the treatment of people with CF. Participation was voluntary and no compensation was provided to the participants.

## Results

### Demographics

A total of 81 people with CF gave informed consent to participate in this study. Of all participants, 89% (72/81) of people with CF and 65% (24/37) of HCPs responded to the questionnaires. The full questionnaire results are presented in Multimedia Appendix 3. We randomly invited 21 people with CF from the questionnaire respondents to participate in semistructured interviews. Due to the recruitment procedure of HCPs for interviews, the total number of invited HCPs was unknown. A subgroup of 12 people with CF and 7 HCPs participated in semistructured interviews until data saturation was reached. The demographics of the participants are presented in Table 1.

**Table 1:** Demographics of participants.

				Questionnaire respondents		Interviewees
**People with CF^a^ (n=81), n (%)**						
	Overall			72 (89)		12 (15)
	Sex (male), n (%)			34 (47)		5 (42)
	Age (y), median (IQR)			31 (15.5-43.5)		15 (10.75-37.25)
	**Age distribution (y), n (%)**					
		6-12	8 (11)		3 (25)	
		12-18	12 (17)		4 (33)	
		18-30	14 (19)		1 (8)	
		≥30	38 (53)		4 (33)	
	Age (y), range			8-61		8-53
	**Hospital, n (%)**					
		Radboudumc	14 (19)		4 (33)	
		HagaZiekenhuis	13 (18)		2 (17)	
		Maastricht UMC+^b^	21 (29)		2 (17)	
		UMC Groningen	11 (15)		2 (17)	
		Amsterdam UMC	13 (18)		2 (17)	
	**CFTR^c^ genotype, n (%)**					
		F508del homozygous	46 (64)		9 (75)	
		F508del heterozygous	22 (31)		3 (25)	
		Other	4 (6)		0 (0)	
	Elexacaftor/tezacaftor/ivacaftor in 2022, n (%)			61 (85)		9 (75)
	Pancreas enzyme use, n (%)			61 (85)		11 (92)
	Months of using the program at recruitment, median (IQR)			21 (19-22)		21 (19.5-22)
**Health care professionals (n=37), n (%)**						
	Overall			24 (65)		7 (19)
	**Specialty, n (%)**					
		Pediatric pulmonology	12 (50)		3 (43)	
		Pulmonology	10 (42)		3 (43)	
		Both	2 (8)		1 (14)	
	**Profession, n (%)**					
		Medical doctor	12 (50)		2 (29)	
		Specialist nurse or nurse practitioner	12 (50)		5 (71)	
	Sex (male), n (%)			3 (12)		0 (0)
	**Age group (y), n (%)**					
		<30	2 (8)		0 (0)	
		30-35	2 (8)		0 (0)	
		35-40	8 (33)		2 (29)	
		40-45	4 (17)		3 (43)	
		45-50	0 (0)		0 (0)	
		>50	8 (33)		2 (29)	
	**Hospital, n (%)**					
		Radboudumc	6 (25)		3 (43)	
		HagaZiekenhuis	3 (13)		1 (14)	
		Maastricht UMC+	5 (21)		2 (29)	
		UMC Groningen	6 (25)		1 (14)	
		Amsterdam UMC	4 (17)		0 (0)	
	Years of work experience, median (IQR)			9 (4-17.5)		10 (1.5-18)

^a^CF: cystic fibrosis.

^b^UMC: University Medical Center.

^c^CFTR: cystic fibrosis transmembrane conductance regulator.

### Qualitative Analyses

Open-ended questionnaire analysis resulted in 28 subthemes. Most questionnaire findings were included in the qualitative part of this study. Findings that were not included either had high agreement rates (eg, no people with CF or parents felt too closely watched by their CF team) or required no further qualitative explanations (eg, the devices that people wanted to be able to access the RMP on). Qualitative analyses of interviews resulted in 380 specific codes divided into 7 themes and 12 subthemes. The themes and subthemes are presented in Multimedia Appendix 6.

### Use Behavior

**Figure 1:**
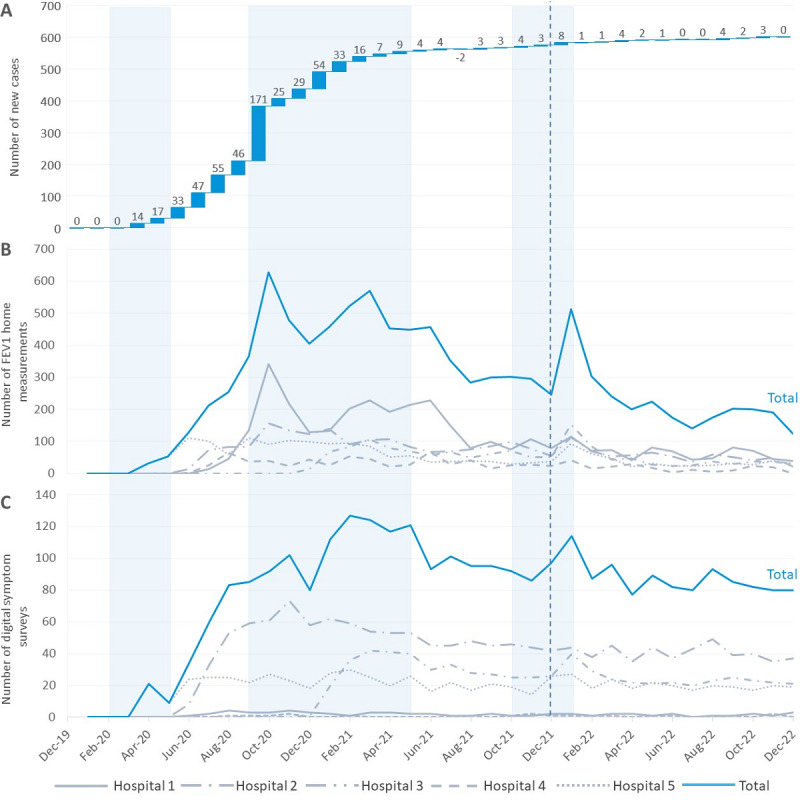
(A) Waterfall plot of the number of new users by month. (B) The number of lung function tests by month. (C) The number of symptom surveys by month. The colored bars represent Dutch COVID-19 lockdown periods. The dashed line represents the introduction of the new modulator drug elexacaftor/tezacaftor/ivacaftor in the Netherlands. FEV1: forced expiratory volume in one second.

Between the second quarter of 2020 and the end of 2022, a total of 608 people with CF were enrolled on the program, and a total of 9418 lung function tests and 2631 symptom surveys were conducted. Questionnaire respondents reported that they used the program weekly (7/72, 10%), monthly (19/72, 26%), during symptoms (28/72, 39%), or rarely (18/72, 25%). Figure 1 shows the monthly enrollment rate, lung function rate, and symptom survey rate for all users enrolled in the program between January 2020 and December 2022. In interviews, HCPs reported a reduction in use frequency over time and especially after the introduction of a new modulator therapy (elexacaftor/tezacaftor/ivacaftor [ETI]). HCPs generally categorized people with CF into 4 different user groups: those who had never started using the program, those who started but discontinued using the program due to difficulties (technical, psychosocial, or otherwise), those who used the program only on indication, or those who used the program regularly. Consequently, HCPs doubted whether the program was suitable for everyone (Textbox 1, quote 1).

**Selection of supporting quotes from interviews translated from Dutch.**

**Quote 1**

- “At first, we gave everyone a spirometer. For some, it quickly ended up in a box somewhere. Others started using it but couldn’t get it to work, so it still ended up in a box somewhere. Quite some patients do actually use it. Some somewhat more diligently at first. I set a reminder for a symptom survey every 14 days and ask patients to also measure their lung function at these moments. Some were really adherent at first, but then couldn’t sustain this pattern but take it up again every now and then. And others remain very adherent. For myself, I let go of the idea that I should set up everyone with these devices [...]. Now, I ask patients more directly whether they actually want to use these devices before I give them one.” (Interviewee 1; health care professional [HCP]).

**Quote 2**

- “The measurement method is different than in the hospital. Different support, different mouthpiece, different technique. And that also means that I don’t always agree with the outcome for 100%.” (Interviewee 2; person with cystic fibrosis [CF]).

**Quote 3**

- “[Low results] don’t really encourage them to keep measuring at home because it is disappointing. We try to motivate patients by explaining that this is a recurring issue, and that it takes time to get used to measuring lung functions at home, and that it will resolve itself [...]. So, we try to make it clear beforehand that there are differences and that the differences will become smaller when they keep practicing [...]. Sometimes this helps, but sometimes it doesn’t” (Interviewee 3; HCP).

**Quote 4**

- “I think that mostly patients themselves really appreciate it [...]. There are different types of patients of course. Some really want to stay in control and want to be able to see how they are doing, and the program is really easy to use for these ends. These patients usually use it well and frequently. There are also patients who still aren’t doing so well and who dislike feeling like a patient and having to go the hospital for a full day. They would prefer being at home to work or something. I think that this program can be used very well as a tool to have check-ups at home. I also think, for myself at least, that there is a third group, those people which I have very little control over and don’t show up to outpatient visits. I hope this program can be an intermediate solution to stay in touch with them” (Interviewee 4; HCP).

**Quote 5**

- “[The benefit], right now, is being able to keep track of my stability. Really being able to see whether the trend is going up or down [...]. Just a type of certainty, which also helps to reduce hospital visits. Now, I can just skip a few and then go again after four months instead of much more often [...]. That is a very big advantage for me, also because I live so far away from the hospital” (Interviewee 5; person with CF).

**Quote 6**

- “We noticed that before we used the program, we felt tense before outpatient visits because of how [my child’s] lung function would be. And we actually had no insight in it. Since we use the program, outpatient visits feel so much more relaxed and pleasant, because we know that everything is fine.” (Interviewee 6; parent of child with CF).

**Quote 7**

- Interviewee: “For some children, but that is a minority, it also gives stress. We withdrew a few patients from the program, because the home measurements gave too much stress. Obsessive amounts of measurements and compulsive parents. The benefits just didn’t outweigh the burden anymore. Maybe we can restart when [their children] are older, but this can also be an effect.”
- Interviewer: “How does this present itself, and where does this stress originate from?”
- Interviewee: “A child blows very variably, because he apparently isn’t skilled enough yet and parents really struggle with this. They think: ‘well this can’t be right.’ They might want their child to blow too often, and at a certain moment the child will think: ‘let it go, I don’t want to anymore.’ Of course the child really needs to have a good technique, so then the interaction between [parents and the child] just isn’t right and then it’s smart to just pause it for a while” (Interviewee 7; HCP).

**Quote 8**

- “I asked [my son]: what do you think about the device yourself? [...] He answered ‘I don’t really like it.’ Then I asked him: ‘And what if it could help you to go to the hospital less?’ Then it was absolutely fine” (Interviewee 8; parent of child with CF).

**Quote 9**

- “How often do I use it? Well, not so often. Once a month, once a quarter. Mostly on moments when I feel like checking myself quickly. And that has two reasons. First, Trikafta [...] immediately led to a drastic decrease of typical CF symptoms like coughing and lots of sputum. As a result, my lung function is not really an issue anymore. So I don’t feel the need to measure my lung function every week. Like, ‘am I doing well or not?’ No, I feel well and I know my lung function is relatively stable. I was ill during last Christmas and during these moments it’s great to be able to check my lung function; like does it have an impact or not? But I use it sporadically. On the one hand, because I feel fine, and on the other hand because I sometimes doubt the reliability” (Interviewee 2; person with CF).

**Quote 10**

- “If I am stable, than I would say I only need to visit the hospital physically every half year. In between, depending on how I am doing, I could have short communications every two to three months [...]. But then you only need to go to the hospital physically every six months [...]. During these visits you can hand in sputum, blood, get a physical examination like blood pressure, that kind of stuff. I imagine that when you are extremely stable that maybe you could even go to the hospital every nine months. And have extra check-ups when you don’t trust how it is going, like, having contact by phone. Then you can still always go to the hospital to hand in sputum or something. I think, both for myself and the health system, this is a very good addition” (Interviewee 10; person with CF).

**Quote 11**

- Interviewer: “How do you feel about [frequent monitoring to predict exacerbations]?”
- Interviewee: “I think [daily measurements] are too much [...] I think it depends on your situation. When you are doing worse than others, then maybe it could give you an advantage, when you can truly detect exacerbations early. But in the case of people who are stable, I don’t think that it really has an added value.”
- Interviewer: “That’s clear. Would it have been different in the period before Trikafta?”
- Interviewee: “Then I would have done it, I think. Yes. If it could predict: ‘now you need to pay attention and possibly take action.’ Yes, then I would have definitely used it” (Interviewee 5; person with CF).

**Quote 12**

- “The group of patients for whom I would find it more difficult [to replace outpatient visits with remote monitoring] are those with a certain rejection of their child’s condition. Those parents who prefer not to talk about their child’s CF, or who would prefer to have as few therapies as possible [...]. The danger is that you lose sight of these parents and patients [...]. The group of parents for whom it would be possible are those who have fully accepted their child’s condition. Those who know that you sometimes have to visit the hospital or to be alert during symptoms, and who also stay alert. That is of course our biggest population, and they know the potential consequences for the future of their children when they deny them care. But you really have to know your patients before you can make this assessment together. And you really don’t want to miscalculate and find out in hindsight that you have lost grip on your patient” (Interviewee 11; HCP).

**Quote 13**

- “Eventually, the gain will be in using online monitoring [to reduce regular outpatient visits]. For some patients this will be really suitable. For others, seeing them in the consultation room adds to their therapy adherence and disease knowledge. So, for some this will definitely be possible. However, this will require a certain ‘transition-management’ for some doctors who have to accept that this is how it is going to be in the future. That our hospital visits are not some sacred towers of strength when we talk about CF management. That really requires a change in mentality” (Interviewee 11; HCP).

### Perceived Ease of Use

Overall, usability was well rated. Most users found the RMP easy to use (HCPs: 16/24, 67%; people with CF: 55/72, 76%; *P*=.42), and almost all respondents thought that most users would be able to quickly learn to use the RMP (HCPs: 23/24, 96%; people with CF: 60/72, 83%; *P*=.17). The most reported negative aspect of the RMP’s usability were technical errors related to the portable spirometer. In questionnaires, people with CF reported that they experienced technical errors sometimes (14/72, 19%), often (7/72, 10%), or very frequently (2/72, 3%). Moreover, 42% (10/24) of HCPs and 29% (21/72) of people with CF had doubts about the reliability of the home spirometry measurements, and 25% (6/24) of HCPs compared to 74% (53/72) of people with CF thought that people with CF used a good lung function technique at home.

In interviews, both people with CF and HCPs reported that home measurements were generally lower than hospital measurements or more variable. The mouthpiece of the home spirometer was often found difficult to use, especially by children. Some people with CF reported missing the support of a pulmonary function technician at home (Textbox 1, quote 2). Others found the home spirometer easy and quick to use and found that good education and experience helped improve technique and interpretation. HCPs reported that technical and reliability issues had caused some people with CF to lose motivation to use the program (Textbox 1, quote 3). For the HCPs themselves, the reproducibility of measurements, the flow-volume loop, and context (eg, increased symptoms, deviation from baseline, or own assessment of technique) were important to interpret results.

People with CF suggested adding percentage predicted values for home spirometry outcomes, and HCPs suggested better integration of the program into existing electronic health records and better education and communication about the program and new developments.

### Perceived Usefulness

Questionnaire results showed that most people with CF and HCPs found the program a good addition to daily CF care (people with CF: 44/72, 61%; HCPs: 21/24, 88%; *P*=.02) or their job (HCPs: 21/24, 88%). Less than half of HCPs felt that the program improved work performance (5/24, 21%), productivity (8/24, 33%), or effectiveness (10/24, 42%). On the other hand, HCPs found that the RMP helped recognize early deterioration of people with CF (16/24, 67%) and gave more insights into people with CF’s disease courses (18/24, 75%). In interviews, HCPs reported that most benefits were present at the patient level rather than at a work performance level (Textbox 1, quote 4). However, HCPs especially appreciated being able to remotely monitor acute deterioration, which facilitated remote triage and adjustment and follow-up of treatments.

A total of 49% (35/72) of people with CF (people with CF: 22/54, 41%; parents of people with CF: 13/18, 72%; *P*=.03) believed that they were able to recognize deterioration sooner than without the RMP, 49% (35/72) believed that they needed less in-person outpatient visits when they were doing well, 36% (26/72) felt more in control, and 35% (25/72) felt more motivated for their treatment. From qualitative analyses, the most important benefits for people with CF included improved understanding of their condition, improved control over their condition, better symptom perception, and reductions in unscheduled and routine outpatient visits (Textbox 1, quote 5). Some parents of people with CF mentioned that the program improved communication with their children about their condition. Benefits varied across people with CF.

### Psychosocial Effects

According to questionnaire results, 17% (12/72) of people with CF experienced stress due to the RMP. Qualitative analyses revealed positive psychosocial effects such as reassurance about health status and less anxiety before outpatient visits (Textbox 1, quote 6). However, for others, the program caused negative psychosocial effects such as increased stress and worries, confrontations between parents and children who disliked home measurements, frustrations with technical errors or bad outcomes, fears of deterioration, increased disease burden, and confrontation with health identities. HCPs were mostly aware of these effects, which sometimes required intervention (Textbox 1, quote 7). Most interviewed people with CF believed that the benefits outweighed the negative effects, whereas others had not experienced any psychosocial effects.

### Intention to Use

In both the questionnaire and interviews, the presence of symptoms was found to be an important incentive to use the RMP regularly. People with CF reported that a future reduction in outpatient visits would be a strong incentive for self-monitoring also during periods of well-being (Textbox 1, quote 8). Other incentives included agreements with and encouragement of HCPs regarding measurement frequency, creating a habit or setting reminders, living far away from the hospital, intrinsic motivation and a perceived necessity for self-monitoring, the fact that measurements are easy and quick to perform, and individually experienced benefits. Ultimately, people with CF wanted an adequate balance between monitoring burden and the benefits of use.

Stable periods were accompanied by little motivation for frequent self-monitoring. For many, these stable periods were more prevalent after initiation of ETI. Other disincentives for regular self-monitoring included having a good symptom perception, unclear implementation and goals of remote monitoring, the existence of other communication media (eg, direct phone lines or email), little intrinsic motivation, other health priorities, technical errors or doubts about reliability of outcomes, remote monitoring being mostly supplemental to regular care without reductions in therapy burden elsewhere, and the aforementioned negative psychosocial effects (Textbox 1, quote 9).

### Future Use

A total of 79% (19/24) of HCPs and 75% (54/72) of people with CF wanted more care remotely in the future rather than physically when people with CF were feeling well (ie, hybrid care). Although some people with CF wanted to keep using the RMP as supplemental to routine care, many people with CF and HCPs also supported hybrid care in interviews, especially in adult CF care. The most important driver was improvement in people with CF’s condition due to new modulator drugs. Hybrid care was thought to help people with CF reclaim agency over their lives and reduce the burden of hospital visits. However, there was a consensus among interviewed participants that in-person care could not be replaced fully by remote care and that there was a strong need for individualized approaches (Textbox 1, quote 10). In pediatrics, it was mentioned that evaluating the physical and psychosocial development of children was also important during outpatient follow-up and that this might not be fully replaceable by digital care.

Table 2 shows additional remote monitoring functions identified as useful for CF care in questionnaires. Additional remote microbiology assessments was the only repeated suggestion for future additions in interviews. One pulmonologist mentioned that, when additional measurements are required to assess patients remotely, it would be better to see the patient in person. Interviewees showed little support for pulmonary exacerbation prediction models requiring high self-monitoring frequencies since the introduction of ETI, but some acknowledged that they would have used these models before they started ETI (Textbox 1, quote 11).

**Table 2:** Number of respondents who found the following options useful for a remote monitoring program for cystic fibrosis (CF) care^a^.

	HCPs^b^ (n=24), n (%)	People with CF (n=72), n (%)	*P* value	CF group, n (%)		*P* value
				People with CF (n=54)	Parents (n=18)	
Monitoring of lung function	22 (92)	66 (92)	>.99	51 (94)	15 (83)	.16
Monitoring of pulmonary symptoms	17 (71)	26 (36)	.004^c^	19 (35)	7 (39)	.78
A function that alerts people with CF when they are not doing well	15 (63)	20 (28)	.003^c^	14 (26)	6 (33)	.56
A function that alerts HCPs when people with CF are not doing well	13 (54)	32 (44)	.48	21 (39)	11 (61)	.11
Monitoring gastrointestinal symptoms	6 (25)	15 (21)	.78	10 (19)	5 (28)	.50
Tracking dietary requirements from the dietician or pancreatic enzyme use	6 (25)	7 (10)	.08	6 (11)	1 (6)	.67
Tracking physiotherapy exercises or activity (eg, number of steps)	5 (21)	9 (13)	.33	9 (17)	0 (0)	.10
Tracking medication use or side effects	13 (54)	24 (33)	.09	17 (31)	7 (39)	.58
Tracking how people with CF feel with regard to their CF (eg, fear or confidence)	11 (46)	13 (18)	.01^c^	10 (19)	3 (17)	>.99
As little functions as possible	2 (8)	4 (6)	.64	3 (6)	1 (6)	>.99

^a^Proportions of respondents who selected the functions were compared to each other using the Fisher exact test (health care professionals vs people with CF and people with CF vs parents of people with CF).

^b^HCP: health care professional.

^c^Significant difference.

### Prerequisites

Only 54% (13/24) of HCPs reported that it was easy to predict beforehand which people with CF would benefit from these programs. HCPs identified several requirements for remote monitoring in interviews: regular home measurements to maintain baseline values, a reliable lung function technique, motivation of people with CF, adequate technology savviness or (digital) health literacy, and adequate psychosocial coping strategies. For hybrid care, requirements for patients were found to be stricter and included a stable condition, adequate symptom recognition, alertness and responsibility during deterioration, and adequate therapy adherence. Therefore, an important prerequisite to be able to assess eligibility was being well acquainted with patients and maintaining this relationship remotely (Textbox 1, quote 12).

To optimize remote monitoring, requirements for HCPs included a more coaching role, adopting shared decision-making in deciding remote monitoring goals and methods, a change in mindset, including remote monitoring in regular follow-up conversations, clear department policies with a division of tasks and adequate safety nets, better education of (new) employees on remote monitoring, and improved communication between HCPs and developers. In most centers, the availability of motivated specialist nurses was essential as they performed most remote monitoring tasks (Textbox 1, quote 13).

## Discussion

### Principal Findings

This study identified experiences, future perspectives, and use behavior of remote monitoring in CF care. Our results show broad support among both HCPs and people with CF for remote monitoring. Benefits ranged from improved symptom perception to reduced routine outpatient care. Lung function home measurements were most appreciated. Pitfalls included negative psychosocial effects and the potential to lose sight of patients. Multiple incentives or disincentives to use the program were identified, such as the severity of physical symptoms. Future perspectives were centered on hybrid care models, personalized remote monitoring strategies, and balancing experienced benefits and monitoring burden.

A recent study identified similar patient benefits of remote monitoring, such as improved control and symptom recognition [24]. In previous work, we also found these benefits in pediatric asthma [12]. Unfortunately, these benefits have received little attention in the literature, potentially because they have not been systematically identified, lack good objective end points, or are hard to express in financial savings. Nevertheless, these benefits can be supportive to individual patients or parents. Future research should distinguish the varieties of individual patient benefits as well as identify objective end points. One possible example of end points could be personalized electronic patient-reported outcome measures [25].

The literature suggests that between a third and a half of people with CF are eager to replace regular care with remote care [11,26]. In our study, >75% of HCPs and people with CF wanted more remote care and less in-person care during periods of well-being. Presently, a minimum of 4 outpatient visits annually is a quality criterion for CF outpatient care in the Netherlands and according to the European CF Society best practice guideline [27]. Most interviewees considered a minimum of 2 annual outpatient visits feasible and desirable when supplemented with remote monitoring. These findings are in line with those of Hendra et al [28]. Our findings may be explained by the improved physical condition of our respondents as 85% used ETI and by the overrepresentation of adults in our study. Parents and HCPs in pediatrics also encouraged hybrid care models but were more reluctant to reduce follow-up visits because of safety and the specialty’s broader developmental focus. Nevertheless, a reduction of 50% in regular outpatient visits with remote monitoring has already been proven safe and efficient for pediatric asthma [13,14].

An important benefit not previously identified was the ability of people with CF to objectify symptoms at home, which helped guide HCPs and people with CF through periods of deterioration and recovery. Although the reliability of lung function outcomes was frequently questioned, especially by HCPs, adequate training, education, and regular practice could mostly overcome this issue. Consequently, remote monitoring has potential to reduce unscheduled health care consumption (eg, outpatient visits and emergency department visits), but this needs to be studied further.

Up to 20% of questionnaire respondents experienced increased stress from using the program. For some, the psychological burden of home monitoring required intervention. These results are more discouraging than previously reported acceptance rates [5,11,24,29]. This might be due to selection bias or a lack of good psychosocial screening methods. These findings emphasize that HCPs need to make time for education, regular evaluation, and support. This also requires motivation and new digital health skills for HCPs and especially for CF nurses, who played a crucial role in the remote monitoring of people with CF. Moreover, it illustrates that not everyone might benefit from remote monitoring. Patients should be responsible and motivated and have sufficient (digital) health literacy and adequate coping strategies. Importantly, these interventions should not be forced on patients but should be evaluated on a patient-by-patient basis through open communication and shared decision-making. This requires an adequate relationship between patients and HCPs not just at the initiation of remote monitoring but also throughout the complete patient journey, where a long-term relationship is required to evaluate the success of remote monitoring and make adaptions to the individual strategy accordingly. Maintaining this relationship will pose a new challenge in the era of digital health as the nexus between patients and HCPs will be increasingly distant in both time and space.

Up to 40% of respondents only used the program during periods of increased symptoms. People with CF likely prioritize reducing their disease burden as they improve physically with new modulator drugs [8,30,31]. Therefore, people with CF might be disincentivized to regularly self-monitor when it is mostly supplemental to regular care. In addition, similarly to the findings by Simpson et al [32], people with CF identified less monitoring functions as useful than HCPs. These findings were reinforced by the objective observation that home measurement rates declined after COVID-19 lockdowns and after the introduction of ETI. These disincentives offer an explanation for the low monitoring adherence in many studies [5,11,24,29,33]. It also corresponds to the negative change in attitude of people with CF toward frequent monitoring to predict deterioration after starting ETI. Nonetheless, many people with CF have no access to modulator therapies or have disappointing responses, and they might still benefit from future prediction models. Generally, a balance must be struck between individual benefits and the burden of remote monitoring.

### Strengths and Limitations

Our mixed methods design enabled the integration of multiple sources of data from a large group of people with CF. To prevent both interpretation and confirmation bias, 2 experienced qualitative researchers (YE and MH) with different backgrounds (psychology [YE] and nursing sciences [MH]) were involved in the collection and analysis of qualitative data. These researchers were not involved in the RMP. To our knowledge this is the most comprehensive appraisal of remote monitoring in CF within long-term, multicenter care. Recently, there have been increasing calls for studies such as this one [5]. Moreover, the overlap in findings with those of other studies in pediatric and adult asthma suggests that our results translate well to other pulmonary diseases [12,14].

The COVID-19 pandemic was the direct cause for the initiation of the RMP. Hence, the time of implementation was turbulent and not representative of regular CF care before the pandemic. This should be considered in interpreting the results. For example, few HCPs found that the RMP improved their work performance and productivity, but this might also be explained by a preoccupation with COVID-19–related care and managing the overburdened health system. Consequently, the full potential of the RMP might not have been reached as there was little time to create clear remote monitoring strategies and policies even though they were found to be important prerequisites for successful implementation. We initially aimed to include more medical doctors in interviews because we aimed for equal representation of different HCPs involved in the remote monitoring of people with CF. However, data saturation was reached after 7 interviews. Possibly, CF nurses were more likely to respond to interview invitations as their role was crucial in the implementation of remote monitoring in routine care. Finally, because we recruited participants through informed e-consent to our program, we were unable to reach people with CF who stopped using the program. HCPs were added to provide some insights into this population.

### Future Directions

The results of this study can be used to synthesize 4 future directions of remote monitoring in CF care, which are summarized in Textbox 2 (sensible strategies for remote monitoring). These strategies can be used simultaneously and alternately depending on active needs, capacities, and available resources of people with CF, HCPs, and the overarching health system. In more practical terms, this means that the different sensible strategies should be implemented based on what resources are available to support remote monitoring within the local context and what local priorities for digital support of the health system are in place. At a patient level, the sensible strategies should translate to an iterative process of shared decision-making on remote monitoring goals, methods, and evaluation and adaption that evolves alongside the changing and dynamic needs of patients. This requires clear remote monitoring policies and tasks and should foster the continuing relationship between patients and HCPs.

**Sensible strategies for remote monitoring in chronic respiratory diseases.**

- Patient-driven strategies aim to support individual patients. These strategies include improving symptom perception, self-management, and therapy adherence or tracking disease trajectories. Importantly, patients themselves have most control over what, why, and when they monitor. Patient-driven strategies require the least effort from health care professionals (HCPs) and are mostly supplemental to regular care, but they require patients to be motivated and skilled. In addition, these strategies can be used to gain experience with remote monitoring in a small group of users or to provide niche monitoring functions for a minority of users. A challenge for these strategies will be reimbursement. Future research should focus on identifying the variety of possible goals for patient-driven strategies as well as how these strategies can be evaluated and valued.
- Symptom-driven strategies are used to guide patients and HCPs through periods of acute and subacute deterioration. They encompass remote symptom objectification, triage, and the initiation and follow-up of treatments. Symptom-driven strategies are mostly supplemental to regular care and require little system-wide changes but can provide immediate remote point-of-care benefits for both patients and HCPs. Symptom-driven strategies require a minimal monitoring frequency to track baseline values and maintain skills during stable periods. Research should focus on reducing irregular health care consumption, such as emergency department visits and inpatient stays, and on cost-effectiveness.
- Care-driven strategies are used to replace regular care through remote monitoring, whereas symptom-driven strategies have more potential for irregular care. Hybrid care models can potentially improve patient emancipation; reduce health care costs and workload; and, therefore, strengthen health systems. However, care-driven strategies require significant effort from both patients and HCPs, system-wide changes, well-designed policies, and adequate safety nets. Consequently, there is a need for more research into the requirements, cost-effectiveness, safety, and implementation of these strategies.
- Prediction-driven strategies are not yet practice based but have received significant attention in the literature. These strategies distinguish themselves from symptom-driven strategies through frequent, multiparametric monitoring and algorithms designed to predict exacerbations or other clinical outcomes. Prediction-driven strategies might be suitable for patients with an unstable condition and frequent acute and subacute deterioration, but there is a need for the validation of these models as well as of their clinical application and efficacy. Importantly, these strategies need to strike a balance between benefits and burden.

The sensible strategies provide a first goal-based frame to conceptualize remote monitoring in clinical practice while recognizing the need for flexibility in applying these principles to local contexts and individual patients. Consequently, the sensible strategies can also be applied in resource-scarce settings where conventional communication media (eg, telephone or SMS text messages) are more accessible than digital programs. As at least patient-driven and care-driven approaches have been identified in previous work in pediatric asthma, it is probable that the sensible strategies can also be used for other CRDs [12,14].

We recognize that the sensible strategies might not be complete, but it is the first goal-based classification of remote monitoring strategies that originated within routine, long-term CRD care. These strategies have the potential to be a first step in resolving the disconnect between current research efforts and clinical applicability as the strategies allow researchers to align their efforts with a clinically relevant strategy that is accompanied by clinical considerations. Moreover, the strategies will empower patients, clinicians, and policy makers to make informed decisions about the use of remote monitoring within their own contexts. Nevertheless, future efforts are needed to make the sensible strategies more actionable by expanding them, validating them, and defining potential end points while acknowledging the inequities in available resources for digital health worldwide.

### Conclusions

Remote monitoring can offer a range of benefits for HCPs and people with CF at both the individual and collective levels. It is essential to integrate remote monitoring strategies into care models according to local capacities and needs to maximize benefits while ensuring feasibility. Many previous studies have focused on predicting deterioration. These interventions are complex and expensive and require significant long-term effort from patients. Therefore, they will not be beneficial for everyone or always feasible in daily practice. The sensible strategies for remote monitoring in chronic respiratory diseases in Textbox 2 can help facilitate the integration of remote monitoring into the care models of CF and other CRDs as it aims to help clinicians, researchers, and policy makers align remote monitoring with local demands and capacities. Future research should pay more attention to the other sensible strategies as they better correspond to value-based health care and could provide immediate support for patients, HCPs, and the overarching health systems.

## Appendix

Multimedia Appendix 1 STROBE (Strengthening the Reporting of Observational Studies in Epidemiology) checklist.

Multimedia Appendix 2 Details of the remote monitoring program.

Multimedia Appendix 3 Questionnaires including answers (translated).

Multimedia Appendix 4 Interview guides (translated into English).

Multimedia Appendix 5 COREQ (Consolidated Criteria for Reporting Qualitative Research) checklist.

Multimedia Appendix 6 Themes and subthemes.

## Abbreviations

CF: cystic fibrosis
CFTR: cystic fibrosis transmembrane conductance regulator
COREQ: Consolidated Criteria for Reporting Qualitative Research
CRD: chronic respiratory disease
ETI: elexacaftor/tezacaftor/ivacaftor
HCP: health care professional
RMP: remote monitoring program
TAM: technology acceptance model

## Data Availability

Anonymized data from this study will be made available upon reasonable request.
