# Supplementary material for: Potential, Pitfalls, and Future Directions for Remote Monitoring of Chronic Respiratory Diseases: Multicenter Mixed Methods Study in Routine Cystic Fibrosis Care
Source: J Med Internet Res. 2024 Aug 6;26:e54942. doi: 10.2196/54942 (PMC11336494; doi:10.2196/54942)
Supplement: Multimedia Appendix 2 [file jmir_v26i1e54942_app2.docx]

**Appendix 2: Details of the remote monitoring platform**

*Data in images within this appendix are shown with consent from the user and anonymized using fictional identifiable information. Language of all images are in Dutch.*

## General overview

The remote monitoring programme (RMP) ‘Luchtbrug-CF’ (Airlift-CF) is an adaption of a RMP used in regular paediatric asthma care.[12-14] Luchtbrug-CF is used to monitor symptoms and lung function, and to facilitate safe and easy patient-physician contact. Every hospital which uses the RMP received their own ‘digital environment’. These environments can be adjusted to local preferences by the ‘environment owner’.

People with CF (pwCF) who have interest in using the RMP receive an account from their healthcare professional (HCP). After completing registration, pwCF can log-in to the RMP using the website (**Image 1**) or smartphone application (**Image 2**) available from the iOS AppStore or Google PlayStore.

The RMP is free to use for pwCF and portable spirometers for all pwCF were donated by the Dutch Cystic Fibrosis Foundation (NCFS).


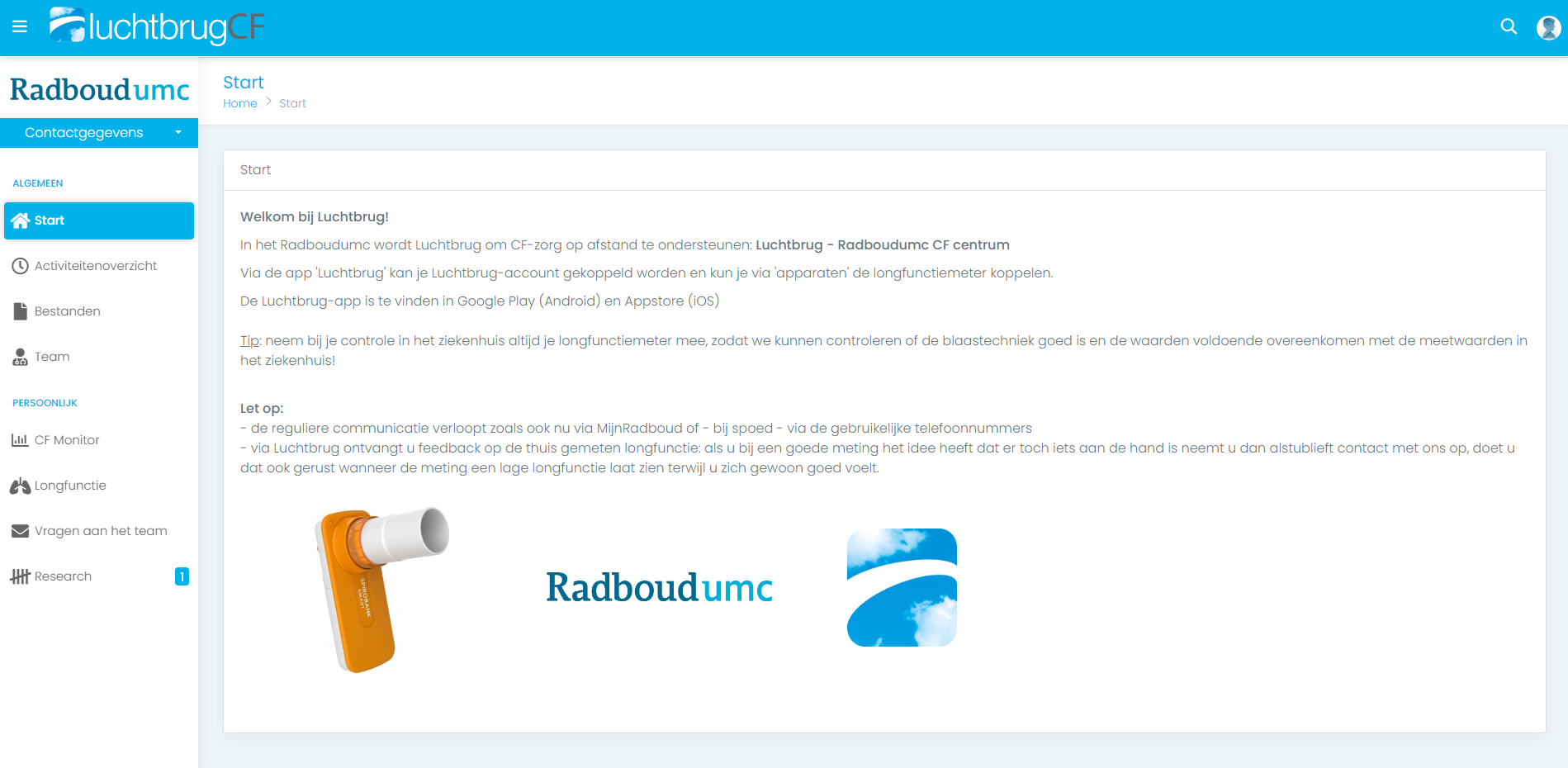


**Image 1.** Overview of the webpage of the RMP.


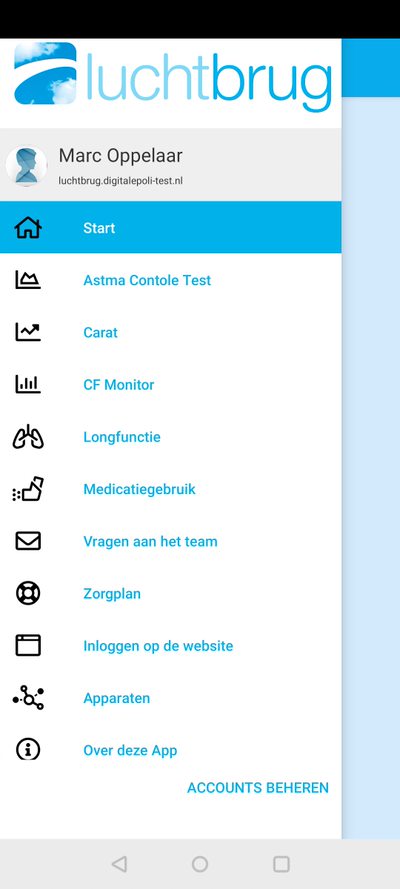


**Image 2.** Screenshot of the smartphone application on Android.

## Lung function tests

Spirometry measurements are performed with the Spirobank Smart and automatically uploaded to the RMP with the smartphone app. After pairing with the spirometer once, users can use the app to perform measurements. Measured values are automatically sent to the app via Bluetooth and can be uploaded directly to the RMP (**Image 3**). The Spirobank Smart device measures forced expiratory volume in one second (FEV1), forced vital capacity (FVC), FEV1-to-FVC ratio (FEV1 /FVC), peak expiratory flow (PEF), and forced expiratory flow at 25%-75% (FEF25-75), and also displays the flow-volume loop. Automated feedback is given for each measurement and is based on FEV1. A green, orange, or red tag is provided based on personalized thresholds depending on the best value of FEV1 of that patient. A green tag prompts encouraging feedback and requires no intervention, while an orange or red tag (i.e. usually below 85% and 70% of the user’s personal best respectively) asked pwCF to self-monitor more often during the next few days until symptoms have resolved, or to contact their healthcare professionals when the patient has concerns. A red tag also sends an automatic email notification to the treating HCPs which they could follow-up on if deemed necessary. FEV1 values are plotted over time in the website and smartphone application (**Image 4** and **5**). The FEV1 is the main lung function outcome on the RMP, but additional values as well and the flow-volume loop are visible when selecting specific measurements on the platform (**Image 6**).


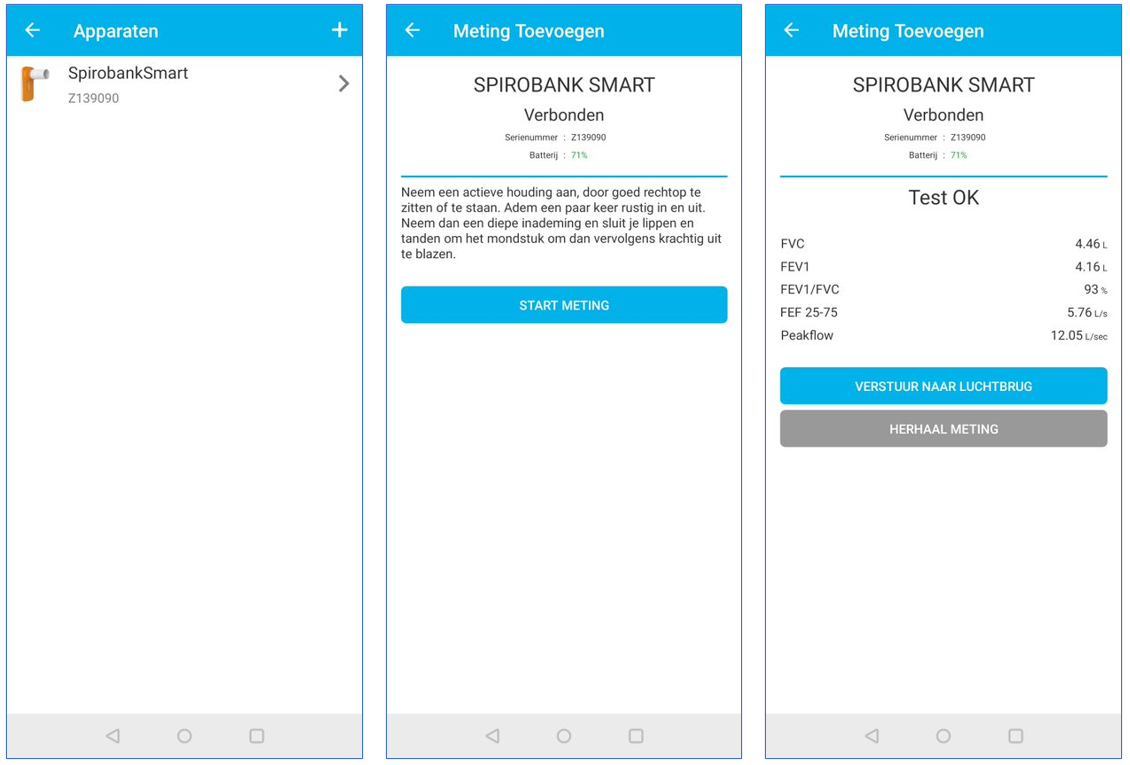


**Image 3:** Screenshots from the Android smartphone application showing user flow for lung function tests. From left to right: selection of coupled lung function device; instructions on how to perform a good lung function test and a button to start the measurement; overview of the results with a button to either upload the measurement or repeat the measurement.


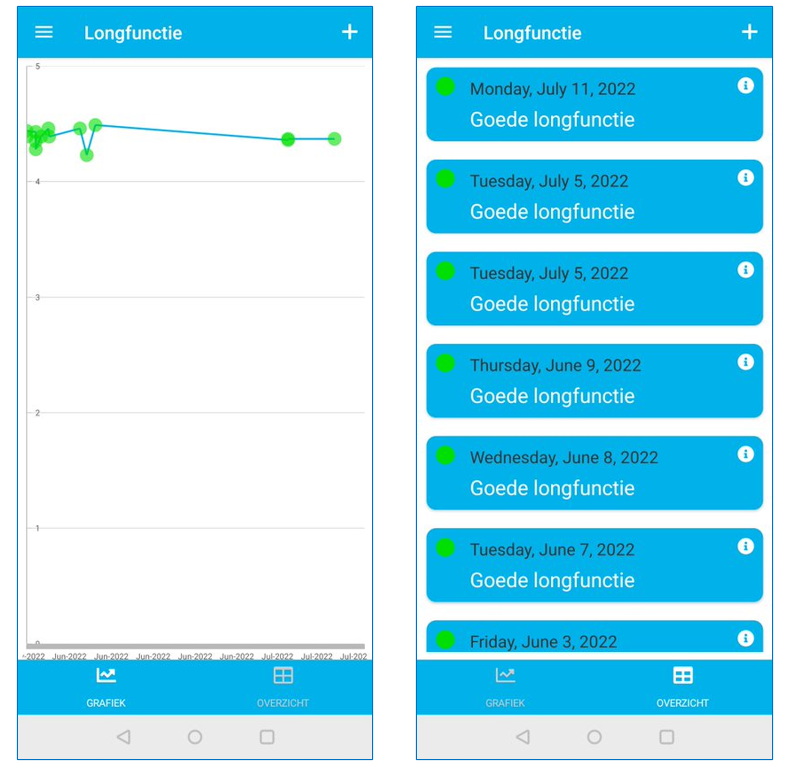


**Image 4.** Screenshot of the Android smartphone application showing the visual presentation of lung function outcomes.


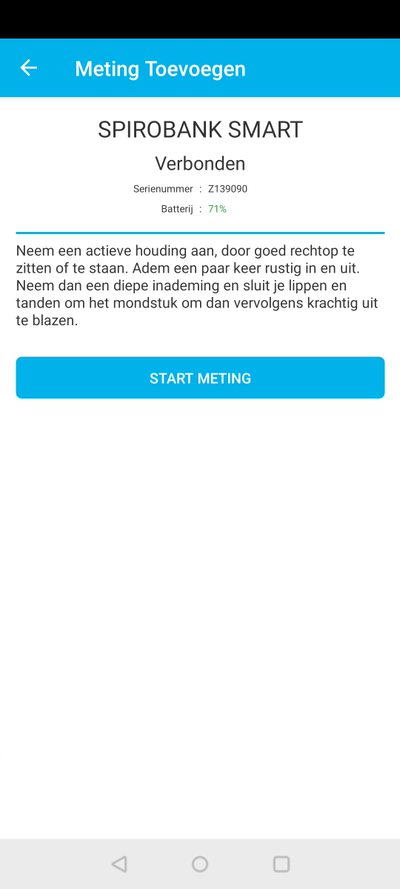

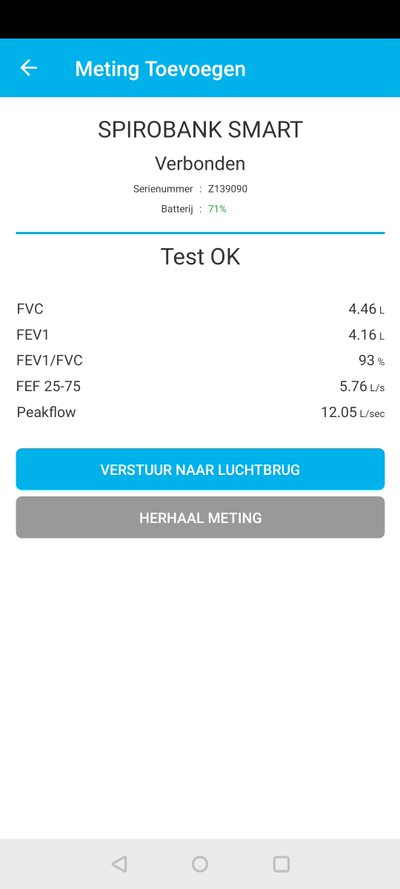

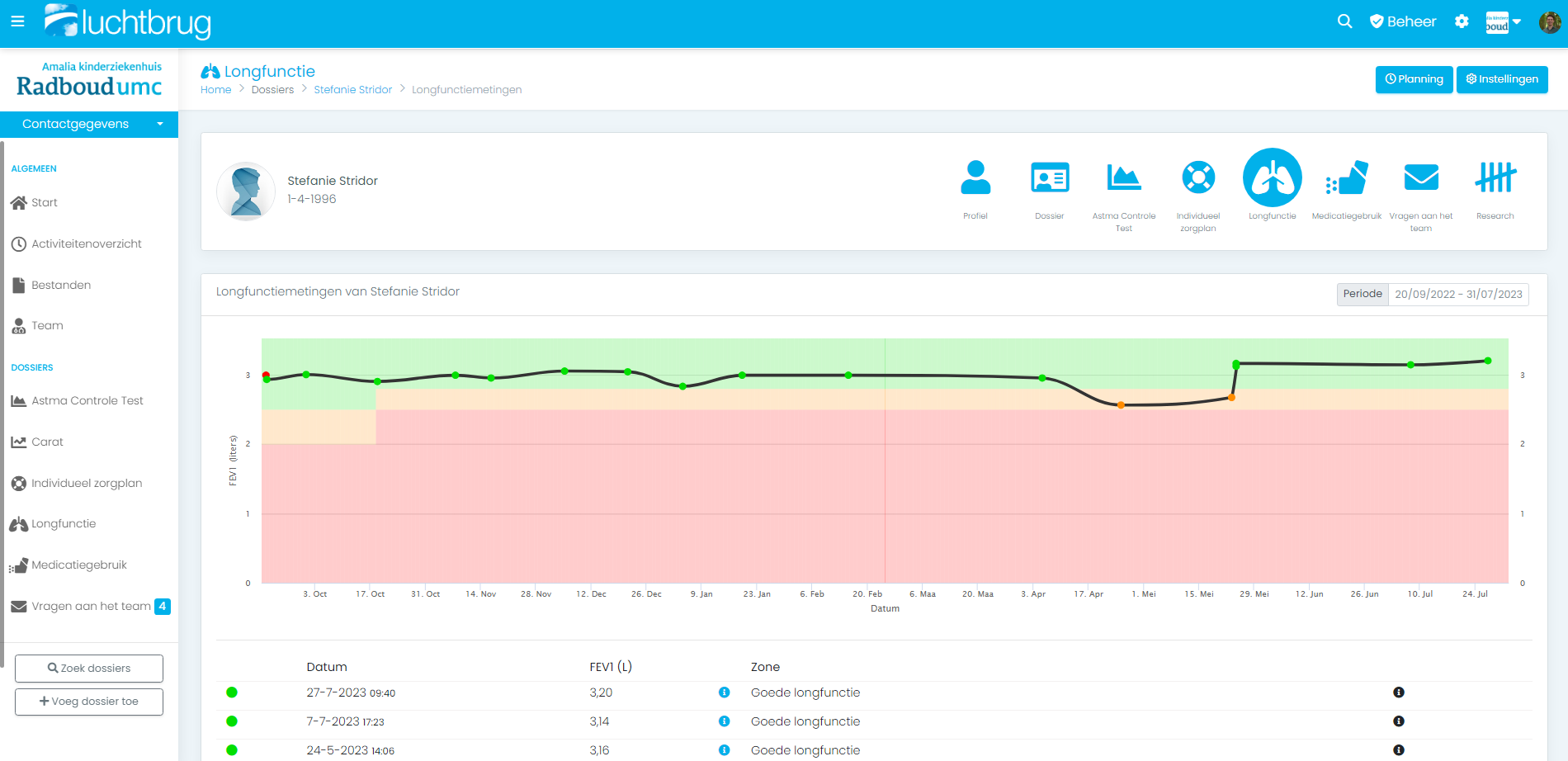


**Image 5**. Desktop screenshot of the graphical visualisation of lung function outcomes.

**
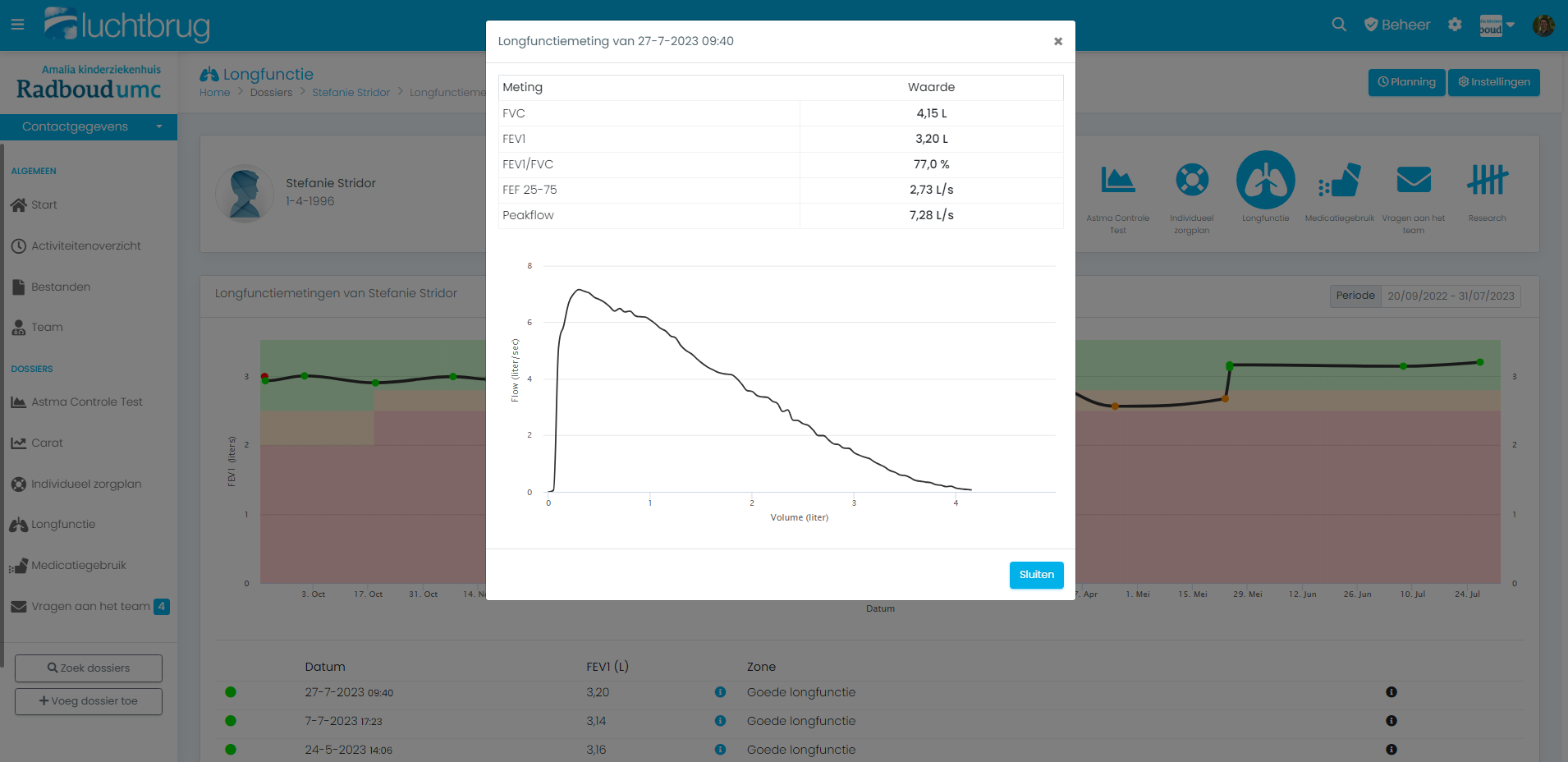
**

**Image 6.** Detailed outcomes of a lung function test including display of the flow-volume loop.

## Symptom surveys

Symptoms are monitored using a questionnaire called the “CF-monitor”. The CF-monitor consists of the 7-item Modified Fuchs Criteria symptom survey. PwCF can fill-out a symptom survey using the smartphone application or website. A result of 6 or larger prompts encouraging feedback, whereas a result of 5 or lower indicated increased symptoms and encouraged the user to self-monitor more frequently the following days, or contact their HCPs. Results of symptom surveys are graphically visualised on the website and smartphone application (Image 7).


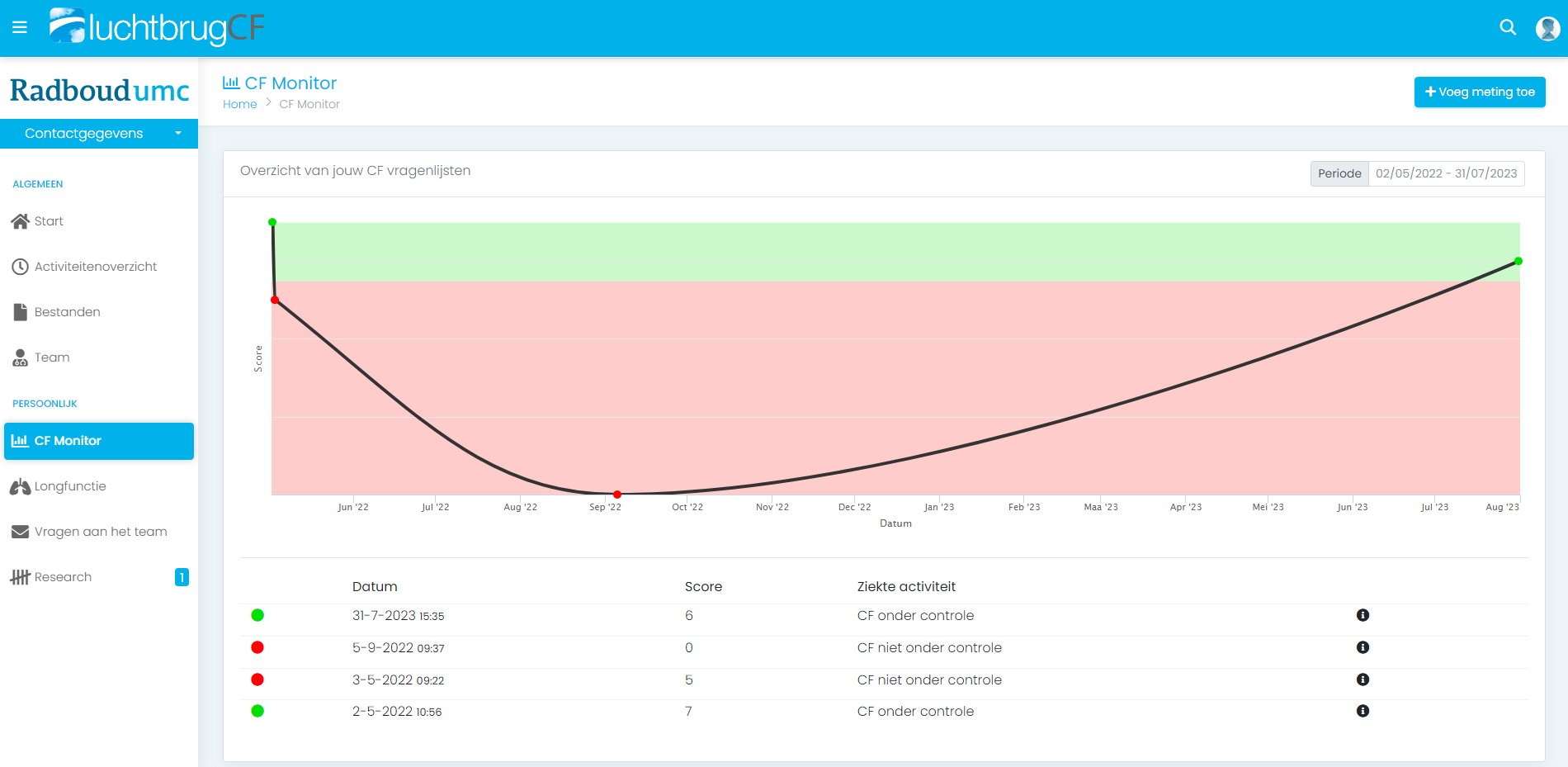


**Image 7.** Desktop screenshot of the graphical visualisation of the CF-monitor on the website. Data presented are fictional and not from an actual user.
