# Supplementary material for: Potential, Pitfalls, and Future Directions for Remote Monitoring of Chronic Respiratory Diseases: Multicenter Mixed Methods Study in Routine Cystic Fibrosis Care
Source: J Med Internet Res. 2024 Aug 6;26:e54942. doi: 10.2196/54942 (PMC11336494; doi:10.2196/54942)
Supplement: Multimedia Appendix 3 [file jmir_v26i1e54942_app3.docx]

**Appendix 3 – Self-designed questionnaires for HCP and pwCF (translated from Dutch)**

**Questionnaire for healthcare professionals including answers (n = 24)**

1. **My gender is:** Male / Female / Other

***Results:*** *see demographics*

1. **My age category is**: <30 years / 30-34 years / 35-39 years / 40-44 years / 45-50 years / >50 years

***Results:*** *see demographics*

1. **I work in the following hospital:** Amsterdam / Maastricht / the Hague / Nijmegen / Groningen

***Results:*** *see demographics*

1. **I treat:** children / adults

***Results:*** *see demographics*

1. **My function is**: medical doctor / specialist nurse or nurse practitioner / other

***Results:*** *see demographics*

1. **I have … years of work experience**: open

***Results:*** *see demographics*

1. **I think it is normal to use digital health programmes like Luchtbrug:** 5-item Likert*

***Results:*** *Agree or strongly agree: 75.0%*

1. **When my patients are doing well, I want more care online and less in the hospital:** 5-item Likert*

***Results:*** *Agree or strongly agree: 79.2%*

1. **I think the following functions would be useful for a digital health programme like Luchtbrug**:^A^

***Results****: see table 3 in manuscript*

1. **A digital health programme like Luchtbrug works best for me when I can use it on**:^B^

***Results:*** *Computer 45.8%, Application 0%, both 50%, no preference 4.2%*

1. **Luchtbrug improves my work performance:** 5-item Likert*

***Results:*** *Agree or strongly agree: 20.8%*

1. **Luchtbrug improves my productivity:** 5-item Likert*

***Results:*** *Agree or strongly agree: 33.3%*

1. **Luchtbrug improves my effectiveness at work**: 5-item Likert*

***Results:*** *Agree or strongly agree: 41.7%*

1. **Luchtbrug is a useful addition to my job:** 5-item Likert*

***Results:*** *Agree or strongly agree: 87.5%*

1. **I can recognize deterioration of my patients sooner thanks to the portable spirometer:** 5-item Likert*

***Results:*** *Agree or strongly agree: 66.7%*

1. **I think my patients can recognize deterioration of themselves sooner thanks to Luchtbrug and the portable spirometer:** 5-item Likert*

***Results:*** *Agree or strongly agree: 75.0%*

1. **Luchtbrug and the portable spirometer give me more insights in the disease courses of my patients:** 5-item Likert*

***Results:*** *Agree or strongly agree: 75.0%*

1. **I think that the treatment of my patients has become more unpersonal due to Luchtbrug:** 5-item Likert*

***Results:*** *Agree or strongly agree: 12.5%*

1. **I think that there is a need for less outpatient visits thanks to Luchtbrug and the portable spirometers:** 5-item Likert*

***Results:*** *Agree or strongly agree: 45.8%*

1. **I think that my patients feel more in control since they use Luchtbrug and the portable spirometers:** 5-item Likert*

***Results:*** *Agree or strongly agree: 29.2%*

1. **I think that my patients think more about their CF than normally since they have started using Luchtbrug and the portable spirometers**: 5-item Likert*

***Results:*** *Agree or strongly agree: 8.3%*

1. **I think that Luchtbrug and the portable spirometers motivate my patients to actively work on their treatment:** 5-item Likert*

***Results:*** *Agree or strongly agree: 25.0%*

1. **I find Luchtbrug a good addition for the daily CF care:** 5-item Likert*

***Results:*** *Agree or strongly agree: 87.5%*

1. **Using Luchtbrug costs me a lot of time:** 5-item Likert*

***Results:*** *Agree or strongly agree: 12.5%*

1. **Keeping up with the administration of Luchtbrug gives me stress:** 5-item Likert*

***Results:*** *Agree or strongly agree: 4.2%*

1. **I can predict well which patients eventually benefit from Luchtbrug:** 5-item Likert*

***Results:*** *Agree or strongly agree: 54.2%*

1. **I have doubts about the reliability of the portable spirometer:** 5-item Likert*

***Results:*** *Agree or strongly agree: 41.7%*

1. **I trust that my patients are able to use a good lung function technique at home:** 5-item Likert*

***Results:*** *Agree or strongly agree: 30%*

1. **I would like to keep using Luchtbrug and the portable spirometer:** 5-item Likert*

***Results:*** *Agree or strongly agree: 91.7%*

1. **I found Luchtbrug unnecessarily complex: :** 5-item Likert*

***Results:*** *Agree or strongly agree: 4.2%*

1. **I thought Luchtbrug was easy to use***:* 5-item Likert*

***Results:*** *Agree or strongly agree: 66.7%*

1. **I think that I would need the support of a technical person to be able to use Luchtbrug***:* 5-item Likert*

***Results:*** *Agree or strongly agree: 8.3%*

1. **I found the various functions of Luchtbrug were well integrated:** 5-item Likert*

***Results:*** *Agree or strongly agree: 37.5%*

1. **I thought there was too much inconsistency in Luchtbrug***:* 5-item Likert*

***Results:*** *Agree or strongly agree: 4.2%*

1. **I would imagine that most people would learn to use Luchtbrug very quickly:** 5-item Likert*

***Results:*** *Agree or strongly agree: 95.9%*

1. **I found Luchtbrug very cumbersome to use:** 5-item Likert*

***Results:*** *Agree or strongly agree: 8.2%*

1. **I felt very confident using Luchtbrug:** 5-item Likert*

***Results:*** *Agree or strongly agree: 66.3%*

1. **I needed to learn a lot of things before I could get going with Luchtbrug:** 5-item Likert*

***Results:*** *Agree or strongly agree: 4.2%*

1. **I regularly agree with the recommendations provided by Luchtbrug:** 5-item Likert*

***Results:*** *Agree or strongly agree: 20.8%*

1. **I know who to contact when I experience problems / have questions about Luchtbrug or the portable spirometer:** 5-item Likert*

***Results:*** *Agree or strongly agree: 79.2%*

1. **My questions and problems are solved quickly:** 5-item Likert*

***Results:*** *Agree or strongly agree: 33.3%*

1. **I think that the portable spirometer looks nice:** 5-item Likert*

***Results:*** *Agree or strongly agree: 37.5%*

1. **I experience technical errors with the lung function device:** Very often/Often/Sometimes/Rarely/Never

***Results:*** *Very often 5.0; Often 30.0%; Sometimes 55.0%; Rarely 10.0%; Never 0%; No answer 16.7%.*

1. **I am satisfied with the portable spirometer**: 5-item Likert*

***Results:*** *Agree or strongly agree: 41.7%*

1. **I think that the Luchtbrug webpage looks nice**: 5-item Likert*.

***Results:*** *Agree or strongly agree: 75.0%*

1. **I give Luchtbrug the grade**: 1-10

***Results****: Median 7.0; 25^th^-ile 6.0 - 75^th^-ile 8.0*

1. **Open questions:**
   1. **I think the biggest advantage of Luchtbrug and the portable spirometer is:**
   2. **I think the biggest disadvantage of Luchtbrug and the portable spirometer is**:
   3. **I have the following remarks or suggestions fur Luchtbrug and the portable spirometers:**

*All 5-item Likert* scales ranged from : Strongly disagree – disagree – neutral – agree – strongly agree

A: (multiple answers are possible)

1. Being able to track lung function.
2. Being able to track experienced pulmonary symptoms.
3. A function which alarms my patients when they are not doing well.
4. A function which alarms me when my patients are not doing well.
5. Being able to track experienced abdominal symptoms.
6. Being able to track dietary requirements from the dietician and the use of pancreas enzymes.
7. Being able to track physiotherapy exercises and/or activity (e.g. amount of steps).
8. Being able to track medication use and/or side-effects.
9. Being able to track how my patients are feeling with regards to their CF (anxiety, confidence, etc.)
10. As little as possible.

B: 1. My computer, using a website. / 2. My smartphone/tablet, using an app. / 3. Both / 4. No preference

**Questionnaire for pwCF including answers (n = 72)**

1. **I am filling out this questionnaire for:** myself / my child

***Results:*** *see demographics*

1. **My / my child’s gender is:** Male / Female / Other

***Results:*** *see demographics*

1. **My / my child’s age is:** open

***Results:*** *see demographics*

1. **I think it is normal to use digital health programmes like Luchtbrug:** 5-item Likert*

***Results:*** *Agree or strongly agree:86.1%*

1. **When I am / my child is doing well, I want more care online and less in the hospital:** 5-item Likert*

***Results:*** *Agree or strongly agree:75.0%*

1. **I think the following functions would be useful for a digital health programme like Luchtbrug**:^C^

***Results****: See table 3 in manuscript*

1. **A digital health programme like Luchtbrug works best for me when I can use it on**:^D^

***Results:*** *Computer 4.2%; Application 56.9%; Both 31.9%; No preference 6.9%*

1. **I can recognize deterioration sooner thanks to the portable spirometer:** 5-item Likert*

***Results:*** *Agree or strongly agree:48.6%*

1. **I think that my / my child’s treatment has become more unpersonal due to Luchtbrug**: 5-item Likert*

***Results:*** *Agree or strongly agree:5.6%*

1. **I think that I need / my child needs less outpatient visits thanks to Luchtbrug and the portable spirometer:** 5-item Likert*

***Results:*** *Agree or strongly agree:75.0%*

1. **I feel more in control and more confident since I use Luchtbrug and the portable spirometer:** 5-item Likert*

***Results:*** *Agree or strongly agree:36.1%*

1. **I am / my child is less absent from work/school since I use Luchtbrug and the portable spirometer:** 5-item Likert*

***Results:*** *Agree or strongly agree: 2.8%*

1. **I think / my child thinks more about my/their CF than normal since I use Luchtbrug and the portable spirometer:** 5-item Likert*

***Results:*** *Agree or strongly agree: 11.1%*

1. **Luchtbrug motivates me to actively work on my / my child’s treatment**: 5-item Likert*

***Results:*** *Agree or strongly agree: 34.7%*

1. **Luchtbrug and the portable spirometer give me / my child stress:** 5-item Likert*

***Results:*** *Agree or strongly agree: 16.7%*

1. **I think Luchtbrug is a good addition to my / my child’s daily care**: 5-item Likert*

***Results:*** *Agree or strongly agree: 61.1%*

1. **I have received enough information from my CF team why it is important to measure my lung function at home**: 5-item Likert*

***Results:*** *Agree or strongly agree: 80.6%*

1. **I have received enough information from my CF team to be able to use Luchtbrug adequately:** 5-item Likert*

***Results:*** *Agree or strongly agree: 87.5%*

1. **I know what to do when my / my child’s lung function is lower than expected**: 5-item Likert*

***Results:*** *Agree or strongly agree: 84.7%*

1. **I often agree with the recommendations given by Luchtbrug:** 5-item Likert*

***Results:*** *Agree or strongly agree: 43.1%*

1. **I would like to keep using Luchtbrug and the portable spirometer:** 5-item Likert*

***Results:*** *Agree or strongly agree: 81.9%*

1. **I found Luchtbrug unnecessarily complex: :** 5-item Likert*

***Results:*** *Agree or strongly agree: 7.0%*

1. **I thought Luchtbrug was easy to use***:* 5-item Likert*

***Results:*** *Agree or strongly agree: 76.4%*

1. **I think that I would need the support of a technical person to be able to use Luchtbrug***:* 5-item Likert*

***Results:*** *Agree or strongly agree: 2.8%*

1. **I found the various functions of Luchtbrug were well integrated:** 5-item Likert*

***Results:*** *Agree or strongly agree: 51.4%*

1. **I thought there was too much inconsistency in Luchtbrug***:* 5-item Likert*

***Results:*** *Agree or strongly agree: 8.3%*

1. **I would imagine that most people would learn to use Luchtbrug very quickly:** 5-item Likert*

***Results:*** *Agree or strongly agree: 83.4*

1. **I found Luchtbrug very cumbersome to use:** 5-item Likert*

***Results:*** *Agree or strongly agree: 8.3%*

1. **I felt very confident using Luchtbrug:** 5-item Likert*

***Results:*** *Agree or strongly agree: 55.5%*

1. **I needed to learn a lot of things before I could get going with Luchtbrug:** 5-item Likert*

***Results:*** *Agree or strongly agree: 5.6%*

1. **I have doubts about the reliability of the portable spirometer:** 5-item Likert*

***Results:*** *Agree or strongly agree: 29.2%*

1. **I feel / my child feels too closely watched by my CF team when they can monitor my lung function results**: 5-item Likert*

***Results:*** *Agree or strongly agree: 0%*

1. **I have concerns about the privacy of my / my child’s data**: 5-item Likert*

***Results:*** *Agree or strongly agree: 2.8%*

1. **I know who to contact when I experience / my child experiences problems or have / has questions about Luchtbrug or the portable spirometer:** 5-item Likert*

***Results:*** *Agree or strongly agree: 81.9%*

1. **My questions and problems are solved quickly:** 5-item Likert*

***Results:*** *Agree or strongly agree: 41.7%*

1. **Using Luchtbrug costs me a lot of time:** 5-item Likert*

***Results:*** *Agree or strongly agree: 5.6%*

1. **I think that the Luchtbrug webpage looks nice**: 5-item Likert*

***Results:*** *Agree or strongly agree: 33.3%*

1. **I think that the portable spirometer looks nice:** 5-item Likert*

***Results:*** *Agree or strongly agree: 37.5%*

1. **I experience technical errors with the lung function device:** Always/Often/Sometimes/Rarely/Never

***Results:*** *Very often 2.8; Often 9.7%; Sometimes 19.4%; Rarely 34.7%; Never 33.3%.*

1. **I give Luchtbrug the grade**: 1-10

***Results:*** *Median 7.*5; 25^th^-ile 6.0 – 75^th^-ile 8.0

1. **I (and my child) use Luchtbrug and the portable spirometer … :** daily/weekly/monthly/only during symptoms/rarely

***Results:*** *Daily 0%; weekly 9.7%; monthly 26.4%; only during symptoms 38.9%; rarely 25%.*

1. **I don’t think it is important to use Luchtbrug and the portable spirometer regularly when I have / my child has few symptoms**: 5-item Likert*

***Results:*** *Agree or strongly agree: 56.9%*

1. **Open questions:**
   1. **I think the biggest advantage of Luchtbrug and the portable spirometer is:**
   2. **I think the biggest disadvantage of Luchtbrug and the portable spirometer is**:
   3. **I have the following remarks or suggestions for Luchtbrug and the portable spirometers:**
   4. **What stimulates you / your child to use Luchtbrug regularly?:**
   5. **What discourages you / your child to use Luchtbrug regularly?:**

* All 5-item Likert scales ranged from : Strongly disagree – disagree – neutral – agree – strongly agree

C: (multiple answers are possible)

1. Being able to track lung function.
2. Being able to track experienced pulmonary symptoms.
3. A function which alarms me when I am / my child is not doing well.
4. A function which alarms me when I am / my child is not doing well.
5. Being able to track experienced abdominal symptoms.
6. Being able to track dietary requirements from my dietician and the use of pancreas enzymes.
7. Being able to track physiotherapy exercises and/or activity (e.g. amount of steps).
8. Being able to track medication use and/or side-effects.
9. Being able to track how I am / my child is feeling with regards to their CF (anxiety, confidence, etc.)
10. As little as possible.

D: 1. My computer, using a website. / 2. My smartphone/tablet, using an app. / 3. Both / 4. No preference
