# Supplementary material for: Potential, Pitfalls, and Future Directions for Remote Monitoring of Chronic Respiratory Diseases: Multicenter Mixed Methods Study in Routine Cystic Fibrosis Care
Source: J Med Internet Res. 2024 Aug 6;26:e54942. doi: 10.2196/54942 (PMC11336494; doi:10.2196/54942)
Supplement: Multimedia Appendix 4 [file jmir_v26i1e54942_app4.docx]

**Appendix 4 – Interview guides for pwCF and HCP (Translated from Dutch)**

**Interview guide for pwCF:**

**Questions/discussion points:**

**Theme 1: perceived ease of use**

- **How do you / you and your child experience the use of Luchtbrug?**
  - How easy or difficult do you / you and your child find Luchtbrug to use? Can you tell more about that?
    - *Consider the following:*
      - *Use of the portable spirometer (e.g. blowing technique),*
      - *Filling out the symptom questionnaire*
      - *The website/smartphone application (i.e. sending or uploading results)?*
- **How do you / you and your child experience using the portable spirometer?**
  - Do you / your child manage to use an appropriate technique at home?
  - Do you trust the lung function home measurements?
    - How does this relate to:
      - The measurement technique at home
      - The portable spirometer device itself
    - Have you spoken with your pulmonary nurse or doctor about this?
    - How can we improve this? What are potential solutions according to you?
- **How do you / you and your child experience using the website/smartphone application?** *(also consider technical errors)*
  - How can we improve our applications? What are potential solutions according to you?
- **Do you / you and your child have the necessary knowledge and skills to be able to use Luchtbrug? Can you elaborate on this?**
  - What would help you / your child to use Luchtbrug?

**Theme 2: perceived usefulness**

- **What influence does using Luchtbrug have on your / you and your child?**
  - Do you experience any positive psychosocial effects from using Luchtbrug, for example:
    - Less tension before an outpatient visit,
    - Feeling more in control,
    - …
  - Do you / you and your child experience any negative psychosocial effects from using Luchtbrug, for example:
    - Stress
    - Insecurities,
    - Obsessive measuring,
    - Confrontation with your child who doesn’t want to self-monitor,
    - …
  - What are the consequences of any negative and/or positive psychosocial effects on your use of Luchtbrug?
    - *Probe for every mentioned effect whether it creates a disincentive or incentive to use Luchtbrug (regularly).*
- **Does Luchtbrug meet your / your and your child’s demands?**
  - How does Luchtbrug meet your / your and your child’s demands?
- **What is the biggest benefit of Luchtbrug for you / you and your child?**
  - What are benefits of Luchtbrug for you / you and your child?
  - Which functions have the most value according to you?
  - Do you think that Luchtbrug is a good addition to you / your child’s daily care?
  - Can you / you and your child recognize deterioration sooner by using Luchtbrug?
- **Can Luchtbrug partly replace outpatient visits?**
  - How do you / you and your child feel about this?
  - Is this possible according to you / you and your child?
    - If not, why not?
    - If so, when?

**For people for whom this is already the case**

- - How do you / you and your child experience this?
  - What are prerequisites according to you / you and your child for replacing hospital visits with online care? What do you / you and your child need for this?

**Theme 3: intention to use**

- **How often do you / you and your child use Luchtbrug?**
- **For whom do you / you and your child use Luchtbrug; for you and your personal goals or for your doctor?**
- **What influences your / your and your child’s use of Luchtbrug?**
  - What influences whether you / you and your child do or do not want to make use of Luchtbrug?
- A general recommendation is to conduct a lung function measurement and fill out the symptom survey at least once a month, but you are in control of your own use frequency.
  - What moves you / your and your child to use Luchtbrug?
  - What determines how often you / you and your child use the portable spirometer?
- **What helps you / you and your child to use Luchtbrug regularly?** *(for example: unstable condition)*
- **How do you / you and your child feel about monthly use of Luchtbrug when your / your child’s condition is stable or you / your child experience few symptoms?**
  - What is a good balance for you / you and your child?
  - Is the importance of the measurements discussed with you / your child? Can you explain yourself why it is important to (regularly) measure your / your child’s lung function?
- **When everything is going well, does Luchtbrug still have an added value for you / your child?** *(think about the CF landscape after Trikafta and future CFTR-modulators*)
- **Do you / you and you child think we should add new functions, or should we limit ourselves to lung functions and symptom surveys?**
  - *Consider possible functions such as …[overview functionalities questionnaire]…, measuring heart rate, respiratory rate, blood pressure etc. for example with a watch?*
  - Would you / you and your child want to choose which functions you use (through discussion with your doctor or nurse)?
  - Would you / you and your child like to use any functions we haven’t mentioned yet?
- **How do you / you and your child view the future of Luchtbrug?**
  - What would you like to add to Luchtbrug?
- **Would you like to talk about any other points we haven’t discussed in this interview?**

**Ending**

- Thank interviewee for participation, stop recording.
- Explain how the results will be used.
- Offer to send transcribed interviews when desired.

**Interview guide for healthcare professionals:**

**Questions/discussion points:**

**Theme 1: perceived ease of use**

- **How do you experience using Luchtbrug?**
  - What do you like and dislike?
- **Do patients experience problems with Luchtbrug, for example, not being able to send results? Or other problems?**
  - Are problems universal or do they differ across users?
  - What are potential solutions to these problems according to you?
- **How do you experience the use of the portable spirometer by patients at home?**

*(Background to this question: questionnaire results showed that satisfaction of healthcare professionals was lower than that of patients)*

- - Do you trust
    - the lung function outcomes that patients measure at home?
    - the measurement technique of users?
    - potential error sensitivity of the portable spirometer?
  - How do you interpret the home measurements as a healthcare professional; how do you interpret a low, or a high measurement, and how do you act on them?
  - Since last summer you can view the FV-curve. Has this changed your trust in the home measurements?
- **Is Luchtbrug integrated in your EHR? What does this look like, or what should this like?**
  - Do you find it necessary that every lung function and questionnaire outcomes are automatically uploaded in the EHR?
  - Do you find it necessary to be able to open the Luchtbrug-health record of a patient within the EHR without opening internet?

**Theme 2: perceived usefulness**

1. **What is for patients the biggest benefit of Luchtbrug? Which benefits do you see for patients?**
   - What are the benefits of Luchtbrug for patients?
   - Which function has the most benefit for patients?
   - Do you think Luchtbrug is a good addition to the daily care of patients?
   - Are patients able to recognize deterioration sooner by using Luchtbrug?
2. **What is the biggest benefit of Luchtbrug for you? Which benefits do you experience for yourself in working with Luchtbrug?**
   - What are benefits for you?

*(Think about: better cooperation between healthcare professionals, better division of tasks, more time to spend on other (more complex) patients, more efficient outpatient clinics, improved time management on the long run)*

- - Which function has the most benefit for you?
  - Do you think Luchtbrug is a good addition to the daily care of patients?
  - Are you able to recognize deterioration sooner by using Luchtbrug, because you can stay up to date of how patients are doing?

1. **Can the amount of outpatient visits be reduced by using Luchtbrug? Is that possible for the whole CF population?**
   - How do you feel about this?
   - Is this desirable?
   - What would be necessary to realise this?
2. **As a healthcare professional, are you aware of any psychosocial effects for users (negative or positive) of using Luchtbrug?**
   - Do you recognize reported negative/positive psychosocial effects of using Luchtbrug, like:
     1. Stress,
     2. Insecurities
     3. Obsessive measurements
     4. Confrontation with children who don’t want to self-monitor
     5. Less tension before outpatient visits?
   - How do you notice this?
3. **What are the implications of these negative and psychosocial effects on the use of Luchtbrug according to you?**
4. **Do you think that Luchtbrug is suitable for every patient?**
   - What does a user (patient and/or parent(s)) need to be able to do to make use of Luchtbrug?
   - Who decides whether a user (patient and/or parent(s)) is suited for Luchtbrug?
   - Should Luchtbrug be suitable for every user?
     1. If so, what should this look like?
     2. How do we realise this?
5. **What do you think about the specific recommendations that patients automatically receive in Luchtbrug after a bad measurement or during a lot of symptoms? These recommendations encourage users to increase monitoring frequency during the following period or to get in touch with their CF team.** *(Background to this question: in questionnaires only 25% of healthcare professionals agreed with these recommendations)*
   - Do these recommendations work? Why do you / don’t you agree with these recommendations?
     1. Does monitoring frequency increase after a bad measurement of many symptoms?
     2. Do users get in touch with their CF team, or does the CF team have to get in touch themselves?
   - Do you believe that the recommendations lead to the desired result?

**Theme 3: intention to use**

1. **How often (frequency) do you want to receive data/measurements of patients as a healthcare professional and why?**
   - How do you discuss this with patients?
   - How do you succeed to motivate patients to measure their lung function [with desired frequency] at home?
   - How do you feel about asking patients to use Luchtbrug monthly even though they are not experiencing any symptoms? Should we ask that from patients?

*(Background to this question: 57% of users reported that they didn’t feel it was necessary to regularly measure a lung function when they were feeling well. This creates a dilemma as patients with a good condition and regular monitoring are eligible to reduce outpatient visits.)*

- - According to you, what is the limit of measurement frequency?
  - Is it necessary to improve use frequency of Luchtbrug?
    - If so, how should we do that?
    - If not, why not?

1. **Many people with CF will improve physically with new CFTR modulators like Trikafta, how should we deal with this in the context of digital care (Luchtbrug)?**
   - Do you think that digital care (Luchtbrug) can play a role in the CF care after Trikafta and other future CFTR-modulators?
   - If so, what role? What is your view on this?
2. **In the questionnaire, a few functions were described as potentially useful for Luchtbrug: for example …*[overview functionalities questionnaire]*… Healthcare professionals identified more functions as useful than patients and wanted to monitor more functions than users found acceptable. Do you recognize this?**
   - Do you think that we should add functions or is the lung function test enough?
   - Which data are (additionally) necessary according to you?
     1. Do you know any functions which you would like to use and which have not been mentioned by us?
     2. What happens with the data? *(Measuring just to measure or because it is useful for the patient)*
   - What do you think would be a good balance between the amount of functions and the burden for patients?
3. **How do you perceive the future of Luchtbrug?**

- **Would you like to talk about any other points we haven’t discussed in this interview?**

**Ending**

- Thank interviewee for participation, stop recording.
- Explain how the results will be used.
- Offer to send transcribed interviews when desired.
