# Supplementary material for: Potential, Pitfalls, and Future Directions for Remote Monitoring of Chronic Respiratory Diseases: Multicenter Mixed Methods Study in Routine Cystic Fibrosis Care
Source: J Med Internet Res. 2024 Aug 6;26:e54942. doi: 10.2196/54942 (PMC11336494; doi:10.2196/54942)
Supplement: Multimedia Appendix 6 [file jmir_v26i1e54942_app6.docx]

**Appendix 6 – Themes and subthemes from qualitative questionnaire and interview analyses**

**Themes from open-ended questionnaire analysis**

1. Benefits

1.1 Benefits for people with CF

1.1.1 Lung function measurements at home

1.1.2 Monitoring of health status

1.1.3 Less hospital visits

1.1.4 Continuous monitoring by healthcare professionals

1.1.5 Positive psychosocial effects

1.2 Benefits for healthcare professionals

1.2.1 Monitoring patients remotely

1.2.2 Online check-ups in between hospital visits

1.2.3 Improved insights in patients’ condition

1.2.4 Quantifying symptoms with home spirometry

1.2.5 Less hospital visits

2. Disadvantages

2.1 Disadvantages for people with CF

2.1.1 Reliability of portable spirometer

2.1.2 Lung function technique at home

2.1.3 Technical issues

2.1.4 Negative psychosocial effects

2.2 Disadvantages for healthcare professionals

2.2.1 Technical issues

2.2.2 Reliability of portable spirometer

2.2.3 Integration in existing systems

2.2.4 Lung function technique at home

2.2.5 User suitability

2.2.6 Psychosocial effects for users

3. Suggestions for the future

3.1 Suggestions of people with CF

3.1.1 Improvements of the home spirometry technique

3.1.2 Different units for home spirometry outcomes

3.1.3 Improvements of the reliability of the home spirometer

3.1.4 Improvements of usability

3.2 Suggestions of healthcare professionals

3.2.1 Flow-Volume loop

3.2.2 Integration in existing systems

4. Intention to use for people with CF

4.1 Incentives for people with CF to use the remote monitoring platform

4.2 Disincentives for people with CF to use the remote monitoring platform

**Themes from qualitative analysis of interviews**

1. Perceived Ease of Use (TAM)

1.1 Perceived ease of use of the remote monitoring platform

1.2 Perceived ease of use of the portable spirometer

2. Perceived Usefulness (TAM)

2.1 Perceived usefulness for people with CF

2.2 Perceived usefulness for healthcare professionals

3. Psychosocial effects

3.1 Positive psychosocial effects

3.2 Negative psychosocial effects

4. Intention to Use (TAM)

4.1 Incentives to use the remote monitoring platform

4.2 Disincentives to use the remote monitoring platform

5. Use behaviour (TAM)

6. Future directions

6.1 Future directions for remote monitoring in CF

6.2 Future functions for the remote monitoring platform

7. Prerequisites for remote monitoring in CF

7.1 Prerequisites for people with CF

7.2 Prerequisites for healthcare professionals and systems
